# Uncommon Cause of Psychotic Behavior in a 9-Year-Old Girl: A Case Report

**DOI:** 10.1155/2012/358520

**Published:** 2012-12-18

**Authors:** Willemina K. Van Putten, Said Hachimi-Idrissi, Anna Jansen, Viola Van Gorp, Luc Huyghens

**Affiliations:** ^1^Paediatric Intensive Care Unit, Brussels University Hospital, Laarbeeklaan 101, 1090 Brussels, Belgium; ^2^Department of Emergency Medicine, Ghent University Hospital, De Pintelaan 185, 9000 Ghent, Belgium

## Abstract

Anti-*N*-methyl-*D*-aspartate receptors encephalitis (ANMDARE) is a well-defined, life threatening, but treatable disorder that often occurs as a paraneoplastic manifestation of ovarian teratomas in adult women. We report a child with this disorder who displayed a subacute onset of delirium, seizures, and autonomic instability. Antibodies against NMDA receptor were detectable in the serum and in the cerebrospinal fluid. No teratoma or other tumour was detected. We speculate that the previous viral/mycoplasma infection may be the trigger of this encephalitis. This patient showed a reversal of the neurological symptoms after intravenous immunoglobulin. Prompt recognition of this disorder followed by immunotherapy results in full neurological recovery.

## 1. Introduction

Acute childhood encephalitis is a potentially devastating illness with an incidence of 10.5 cases per 100,000 child years in developing countries [1].

Among encephalitis, encephalitis with antibodies against *N*-methyl-*D*-aspartate-(NMDA-)type glutamate receptors is a well-recognized and well-defined clinical entity. The initial reports of this encephalitis concerned first exclusively young women with ovarian germinal tumour and encephalitis [2–6]. The outcome after tumour resection was favourable [2–6].

The exact incidence of anti-*N*-methyl-*D*-aspartate receptor encephalitis (ANMDARE) is still unknown, but on basis of literature data, this occurs in 4% of patient with encephalitis in United Kingdom [6].

All had a subset of clinical symptoms such as catatonia, seizures, abnormal movements, and limbic encephalitis [7], but the spectrum of the disease is probably much wider than previously thought. Other authors stated that this encephalitis occurs in patients without teratoma and that men as well as children are affected [8]. 

We report here a case of sub-acute-onset encephalitis with anti-NMDA-receptor antibodies in the absence of teratoma in young female child.

## 2. Case Report

A 9-year-old shy and timid girl with no medical history presented with generalized tonic clonic seizures and an altered mental state. Three weeks before admission, the patient had some flu-like symptoms that resolved spontaneously after a period of four days. Two days before admission, she displayed an abnormal behaviour such as disinhibition (stripping and nudity while visitors at home) and verbal aggressiveness toward the parent. During the night she was agitated and continuously talking while sleeping. The morning of the admission she was found comatose and having generalized tonus and clonus movement, had a tongue bite, and was incontinent. When the seizure had stopped, the patient woke up and the family as well as the paramedics noticed an aggressive behaviour and speech dysfunction.

Upon admission she showed an epileptic status with generalized tonic and clonus movements of the all body, which was stopped by intravenous administration of midazolam first followed by diphantoine and later on with continuous sodium valproate because of persistent epileptic insult. When she woke up, she was responsive but displayed an incoherent language. The neurological and the physical exams were normal, except the presence of a few multi-lobed cutaneous vesicles on the patient's back. A CT scan of the brain was normal. A spinal tape was performed which was slightly traumatic with an increased red blood cells count (247/mm^3^, normal range <5), a mild pleocytosis (198/mm^3^, normal range <2) with 78% lymphocytes and 3.5% atypical lymphocytes, elevated lactate (2.3 mmol/L, normal range <1.7), and mildly elevated protein (41 mg/dL, normal range 16–31). A complete hematological work-up failed to reveal the cause of her symptoms; C-reactive protein was normal (CRP <0.5 mg/L). Because of the seizures, she had abnormal cerebrospinal fluid (CSF) and cutaneous vesicles on the back. An empiric antibiotic therapy (ceftriaxone IV) as well as antiviral treatment (acyclovir IV) was initiated.

Repeated blood and CSF serological analysis remain negative: including herpes simplex virus, *Cryptococcus*, West Nile virus, *varicella zoster*, Lyme disease, and cat scratch disease. Serial brain MRIs were normal and the spinal tape control one week later showed only a slightly increased white blood cell count. An electroencephalogram recording showed a continuous, slow, rhythmic activity in the delta-theta activity, which was consistent with encephalitis and antiepileptic drugs impregnation.

The patient's neurologic condition worsened five days after admission and she developed mood changes, irritability and fluctuation in consciousness, hallucination, expressive dysphasia, and facial dyskinesia, diaphoresis, and choreoathetotic movements. Very short episodes of bradycardia (up to 28 beats/min) alternating with low blood pressure (65/35 mmHg) and sometimes with high blood pressure (167/85 mmHg) were displayed. None of these had needed medical interventions. 

On day 7, *Mycoplasma pneumoniae* serology was positive (a single titter of 1 : 320 as determined by complement fixation (CF) assay), but no *M. pneumonia *was detected in CSF, throat, or blood specimens by means of culture or polymerase chain reaction (PCR). A treatment with clarithromycin for ten days was added to the previous antibiotics and antiviral regiments. The clinical evolution and the symptoms did suspect ANMDARE, and a treatment with immunoglobulin (400 mg/kg/day) on day 10 and this for a consecutive five days was initiated.

Residual serum and CSF samples from the day of admission were sent to Weatherall Institute of Molecular Medicine, John Radcliffe Hospital in Oxford, UK (Professor Vincent) for anti-NMDA receptor antibodies analysis [9]. Anti-NMDA-receptor antibodies were significantly high in both samples (no quantitative value was available, but was categorized as significantly positive). 

Additional investigation including abdominal en pelvic ultrasonography showed no abnormal mass. In the subsequent days, the clinical evolution as well as her social behaviour improved gradually and the patient was sent to a revalidation and rehabilitation centre.

At sixteen-month follow-up, she is still seizure-free and had been successfully weaned off anticonvulsant (sodium valproate) medication. She has returned to school and retained premorbid cognitive function and behaviour.

## 3. Discussion

We present the case of a 9-years-old girl with primary symptoms of neuropsychiatric disorder deteriorating into generalized convulsion, autonomic dysfunction, orofacial dyskinesia, and mutism. This presentation is similar to other cases reported in the literature in adults as well as in children and is consistent with ANMDARE [6, 10–14]. 

Initially reported only in young women with ovarian teratoma, later in patients with thorax tumour [2], and finally, more cases of ANMDARE without tumour were described [11, 12, 15].

Indeed, almost half reported patients by Dalmau et al. [8] and Florance et al. [13] were tumour free. This may be secondary to the involution of the tumour at the diagnosis, less sensitive equipment to detect the primary tumour, inadequate and/or insufficient longitudinal follow-up, and may be because of the young age of the patients at the time of the diagnosis.

The absence of a primary tumour in children may be consistent with limitation in investigations such as trans-vaginal ultrasound and exposure to radiation or may be secondary to other mechanisms that initiate this disorder. In a subgroup of patients, the presence of a tumour that expresses ANMDAR, likely trigger the immune response. In patients without tumour, this may be secondary to the lack of investigation accuracy or related to other trigger mechanism such as viral diseases, which lead to a breach of normal immune tolerance [16].

In our case no tumour has been found; however, the symptoms were preceded by a flu-like prodrome followed by psychiatric features and neurological abnormalities as well as autonomic manifestations consistent with similar cases described elsewhere and more surprisingly the majority of them were children [5, 11, 13, 16]. 

A slightly positive *Mycoplasma pneumonia *serology was found in our case; we might speculate that the possible *Mycoplasma pneumonia *infection has led to a burst of immunological reaction. The major role of our immune system is to recognize and clear infections. Sometimes, the components of the immune system triggered by a prior infection most often in children may react with the patient's own body and causes autoimmune diseases. When this reaction is against the brain, autoimmune encephalitis occurred. This observation among others lends itself to the speculation than ANMDARE is the result of a postinfectious, antibody mediated process as has been described in Sydenham's chorea and other paediatric autoimmune neuropsychiatric disorders associated with streptococcal infections [17–20].

At onset, the most distinctive features include prominent psychiatric symptoms with seizures, confusion, and memory loss. Patients will sometimes show bizarre and often rather disturbing behaviour. Typically a few days later, patients develop a movement disorder, fluctuations in blood pressure, heart rate, and temperature, and may have a reduction in their level of consciousness. The movement disorder often consists of continuous writhing and twitching of face and limbs but can also be a generalized slowing down of movements.

The phenotype of the disease course may differ in children compared to adult. There are in fact differences in tumour association, neurological presentation, and frequency of symptoms. It seems that the autonomic manifestation in children appears to be less severe than in adults. Children exhibit less central hypoventilation requiring mechanical ventilation [8]. However, other autonomic manifestations such as urinary incontinence, sleep dysfunction, episodes of hypertension, tachycardia or hyperthermia, and agitation were frequently observed in children [5, 13, 14]. This difference between adult, and children manifestation remained unclear, whether this is related to a difference in the brainstem control, the potential triggering agent, or difference in NMDA receptor expression [5]. On the contrary, psychosis manifestation in children is quite challenging for the parent as well as for the health care providers. Initial symptoms such as temper tantrums, behavioural change, agitation, and progressive speech deterioration can initially be overlooked in children. Other symptoms such abnormal movements and stereotyped motions may be erroneously mistaken for seizures [11, 13, 14].

The clinical features of ANMDARE are distinctive and are prompting many clinicians to request the NMDA receptor-antibody test to diagnose this condition. Once the diagnosis has been established, an underlying tumour should be excluded. This tumour is most commonly an ovarian teratoma. Other causes should also be excluded (particularly infections). In case of positive NMDA receptor antibody, treatments should be initiated. Treatments consist of immunotherapies (such as steroids, immunoglobulin, and plasma exchange) and removal of a tumour, if present. Prompt therapies offer a good chance of substantial recovery in the majority of patients [13].

In our patient, immunotherapy treatment for 4 days was successful. The symptoms improved gradually and revalidation help patient's recovery up to the pre-disease physical and psychological status.

In summary, ANMDARE is an antibody-mediated disease that causes psychiatric features, confusion, memory loss, and seizures followed by a movement disorder, loss of consciousness, and autonomic fluctuations. These symptoms could initially mislead the parents to seek help, and the physician to recognize this disorder. An accurate diagnosis, initial stabilization, and immunotherapy are life saving.
